# Putative involvement of sirtuin modulators in LPS-induced sickness behaviour in mice

**DOI:** 10.1007/s11011-022-00992-9

**Published:** 2022-05-12

**Authors:** Manas Kinra, Niraja Ranadive, Jayesh Mudgal, Yuqing Zhang, Anusha Govindula, Shailendra Anoopkumar-Dukie, Andrew K. Davey, Gary D. Grant, Madhavan Nampoothiri, Devinder Arora

**Affiliations:** 1 Department of Pharmacology, Manipal College of Pharmaceutical Sciences, Manipal Academy of Higher Education (MAHE), Manipal, 576104 India; 2 School of Pharmacy and Medical Sciences, Griffith University, Gold Coast campus, Gold Coast, Queensland 4222 Australia

**Keywords:** Sickness behaviour, SIRT, Resveratrol, Sirtinol, Neuroinflammation, Cytokines

## Abstract

NAD^+^—dependent histone deacetylases (sirtuins 1–7) have been shown to be involved in various pathophysiological conditions including their involvement in cardiovascular, cancerous, neurodegenerative, immune dysregulation and inflammatory conditions. This study investigates the inflammomodulatory potential of resveratrol (RES), a sirtuin activator and sirtinol (SIR), a sirtuin inhibitor in lipopolysaccharide (LPS)-induced model of sickness behaviour in mice. Male Swiss albino mice were divided into five groups (n = 6) consisting of saline (SAL), LPS, RES, SIR, and fluoxetine (FLU) respectively, each group except LPS was prepared by intraperitoneally (i.p.) administration of SAL (10 mL/kg), RES (50 mg/kg), SIR (2 mg/kg) and FLU (10 mg/kg). Thirty minutes after the treatments, all the groups, except SAL were administered LPS (2 mg/kg, i.p.). The behavioural assays including, open field test, forced swim test, and tail suspension tests were conducted 1 h after LPS challenge. LPS administration significantly reduced the locomotor activity along with inducing a state of high immobility and that was prevented by pretreatment with RES and SIR. Further, various proinflammatory cytokines (TNF-α, IL-6, and IL-1β), and oxidative stress markers (MDA and GSH) were found to be significantly elevated in the brain homogenates after LPS treatment. SIR pretreatment abrogated the LPS-induced neuroinflammatory and oxidative stress changes, whereas RES was only effective in reducing the oxidative stress and TNF-α levels. The results of this study speculate that the role of SIRT modulators in neuroinflammatory conditions could vary with their dose, regimen and chemical properties. Further studies with detailed molecular and pharmacokinetic profiling will be needed to explore their therapeutic potentials.

## Introduction

Neuroinflammation is one major underlying cause of several CNS-related diseases (DiSabato et al. 2016). Lipopolysaccharide (LPS), an endotoxin that acts as a Pathogen-Associated Molecular Pattern (PAMPs) and binds to the Toll-like Receptors (TLRs) and triggers neuroinflammatory response. In animal models of neuroinflammation, LPS is administered peripherally, and it leads to an acute sickness behaviour followed by depressive-like state in a biphasic manner (Basu Mallik et al. 2016; 2021; Moraes et al. 2017). Acute sickness triggered by proinflammatory cytokines (IL-1, IL-6 and TNF-α) in response to PAMPs, is therefore an organized strategy to counteract infecting pathogens (Dantzer 2009). Thus, a phenomenological overlap is seen between sickness behaviour and early stages of clinical depression due to the circulatory cytokines (Maes et al. 2012).

Amongst the mammalian histone deacetylases (HDACs), HDAC1-11 are classified as classical zinc-dependent, and HDAC1-7 as nicotinamide adenine dinucleotide (NAD^+^)-dependent sirtuins (SIRT) (Lugrin et al. 2013). HDACs are further sub-grouped into various classes, and they catalyse the cleavage of acetyl groups from lysine residues (Lugrin et al. 2013). Deacetylation of histones causes gene repression, and modulates various non-histone proteins (PPARγ, PGC-1α, p53, NF-κB, p38 MAPK, FOXO1 and FOXO3). It also affects various biological and pathological processes involved in neurodegenerative, auto-immune, cardiovascular and oncologic conditions (Shakespear et al. 2011; Lugrin et al. 2013; Paraíso et al. 2013; Jęśko et al. 2017). Along with the known clinical anticancer properties, classical HDAC inhibitors have also been shown to possess anti-inflammatory and immunomodulatory activities (Shakespear et al. 2011; Jenke et al. 2021).

In vitro data suggests that SIRT1 activation by resveratrol (RES) reduces neuroinflammation by decreasing the levels of IL-1β, IL-6, matrix metalloprotein-9 and iNOS along with decreased acetylated p53 and cleaved caspase 3 (Zhang et al. 2020). Furthermore, chronic RES treatment reversed chronic unpredictable mild stress (CUMS)-induced protein changes leading to increased expression of SIRT1, p-CREB, CREB, and BDNF while reduced miR-134 levels (Shen et al. 2018). Moreover, being a polyphenolic compound RES has been well documented to reduce oxidative and nitrosative stress markers ((Palsamy and Subramanian 2011; Jing et al. 2013; Gordish and Beierwaltes 2014; Park and Pezzuto 2015).

On the other hand, silencing of SIRT2 in microglia reduced the LPS-induced microglial activation thereby lowering the TNF-α and IL-6 levels (Chen et al. 2015). Similarly, LPS-induced ROS generation and NF-κB activation was shown to be significantly reduced in SIRT2 knockout mice (Lee et al. 2014). Moreover, the CONVERGE consortium (CONVERGE consortium 2015) has linked the SIRT1 gene with major depressive disorder. Further studies substantiate that after chronic social defeat stress, SIRT1 expression increases. and administration of RES directly into the nucleus accumbens also activates SIRT1 and produces a phenotype with increased anxiety and depression-like behaviour (Kim et al. 2016).

Interestingly, both sirtinol (SIR), and RES are polyphenolic in structure and can act on multiple cellular targets including steroid-hormone mediated pathways and xenobiotic metabolisms (Wang et al. 2013). The polyphenolic structural similarities between these two compounds could be responsible for an overlap in their biological activities. Based on this background we chose to compare the effects of both RES and SIR on LPS-induced psychopharmacological parameters and brain cytokines which are involved in neuroinflammatory conditions.

## Materials and methods

### Animals

Male Swiss albino mice, (8–10 weeks old, 20–30 g) were used in this study and were procured from the inbred strains of Central Animal research Facility (CARF), Manipal Academy of Higher Education (MAHE), Manipal for the study. All the experimental procedures were approved by the Institutional Animal Ethics Committee (IAEC) of Manipal Academy of Higher Education (IAEC/KMC/25/2020 dated 22/02/2020) and were performed in accordance with the guidelines set out in compliance with the National Institutes of Health Guide for Care and Use of Laboratory Animals (Publication No. 85–23, revised 1985). Animals were housed in groups of 6 under controlled laboratory conditions, maintained at 12 h day and night cycle with free access to food and water.

### Chemicals and Reagents

Lipopolysaccharide (LPS) (*Escherichia coli* serotype O111:B4), fluoxetine hydrochloride, 2-thiobarbituric acid (TBA), sodium dihydrogen phosphate anhydrous, disodium hydrogen phosphate anhydrous and trichloroacetic acid were purchased from Sigma-Aldrich (Sigma-Aldrich Co. LLC (St Louis, MO, USA). Resveratrol and sirtinol were procured from Abcam (Abcam plc, Cambridge, UK). All other chemicals used in this study were of analytical grade.

### Drug treatments

Animals were randomised based on the body weights and we allocated into five groups (n = 6). Group 1 served as control (SAL); group 2 as LPS (SAL + LPS); group 3 as resveratrol treatment (RES + LPS), group 4 as sirtinol treatment (SIR + LPS), and group 5 as fluoxetine treatment (FLU + LPS). All the treatments were administered by intraperitoneal (i.p.) route. SAL and LPS groups were administered normal saline at a dose of 10 mL/kg. RES, SIR and FLU groups were treated with RES (50 mg/kg), SIR (2 mg/kg) and FLU (10 mg/kg) respectively. All animals (except SAL group) received a single injection of LPS (2 mg/kg) 30 min after the treatment. Behavioural assays were performed within 1–2 h of LPS administration and were video recorded. Animals were euthanised at 3 h post LPS injections and brain samples were isolated and stored at -80 °C till further analysis. Tissue samples were homogenised using chilled phosphate buffer (0.1 M, pH 7.4) for antioxidant and cytokine level estimations.

### Behavioural assays

A series of behavioural assays were performed, including open field test (OFT) to measure the spontaneous activity, forced swimming test (FST) and tail suspension test (TST), for the measurement of immobility state. All the assays followed the procedures as described earlier (Wang et al. 2013; Basu Mallik et al. 2016; Mudgal et al. 2019). OFT was measured as the number of line crossings and rearing in a plexiglass chamber (30 cm × 30 cm × 90 cm), where the chamber was divided into 9 equal virtual quadrants of 10 cm × 10 cm. FST was assessed by calculating the total time spent by the animal in an immobile state over the 5 min of observational period in a transparent plexiglass cylindrical tank (30 × 20 cm), whereas TST was recorded as the immobility time with the animals individually hung for a period of 5 min at 15 cm away from the nearest surface.

### Estimation of brain cytokine and lipid peroxidation levels

Cytokines, namely, IL-6, TNF-α and IL-1β were estimated using commercially available kits (Invitrogen, California, USA). Lipid peroxidation (LPO) and reduced glutathione (GSH) were performed as detailed in earlier (Sahu et al. 2019; Mudgal et al. 2019). Brain homogenates were incubated with equal volumes of TBA at 90 °C for 10 min. Malondialdehyde (MDA) formed was measured spectrophotometrically at 532 nm. Similarly, GSH was estimated by the absorbance of GSH and DTNB complex at 412 nM. Total protein estimation was carried out using Pierce™ BCA Protein Assay Kit (ThermoFisher Scientific, USA), as per the manufacturer’s instructions.

### Statistical analysis

All data sets were analysed for statistical significance utilising GraphPad Prism 9.0.0 (Graph Pad Software Inc., San Diego, CA, USA). Values are expressed as means ± S.E.M. Experimental groups were compared against the control groups using one-way analysis of variance (ANOVA) followed by Dunnett’s multiple comparison test. A “*p*” value of < 0.05 was considered to be statistically significant.

## Results

### Effect of RES and SIR on behavioural parameters

**Fig. 1:**
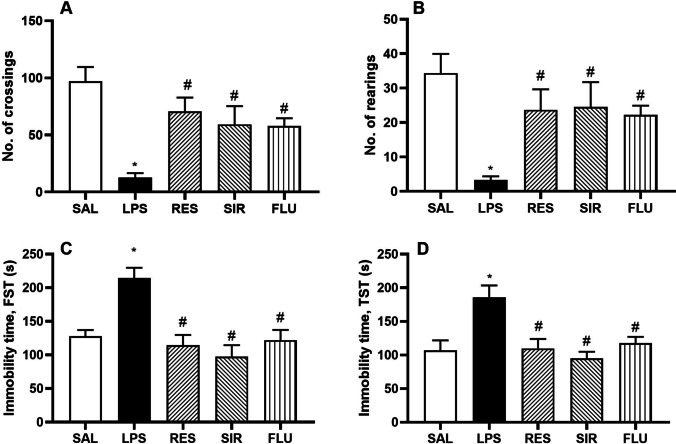
Effect of saline (SAL), resveratrol (RES; 50 mg/kg), sirtinol (SIR; 2 mg/kg) and fluoxetine (FLU; 10 mg/kg) on LPS-induced behavioural changes. Number of crossings (1A); number of rearing (1B); immobility time (s) (FST) (1C); immobility time (s) (TST) (1D). *p < 0.05 as compared to SAL group; #p < 0.05 as compared with LPS group

Administration of LPS produced a significant reduction in the locomotor activity (LMA) as assessed by the number of crossings (12.67 ± 3.89 vs 97.00 ± 12.53 of SAL treated group; Fig. 1A), and number of rearing (3.33 ± 1.02 vs 34.33 ± 5.59 of SAL treated group; Fig. 1B). Pretreatment of the animals with RES, SIR and FLU (70.50 ± 12.30; 59.33 ± 15.94 and 57.83 ± 6.83 respectively, F [4, 25] = 7.46, p < 0.05, Fig. 1A) significantly reduced the LPS-induced effect on LMA and rearing (23.67 ± 5.98; 24.50 ± 7.22; 22.17 ± 2.75 respectively, F [4, 25] = 4.98, p < 0.05, Fig. 1B).

In FST, LPS administration led to a significantly increased immobile state in all the animals (214.30 ± 15.42 s, vs 128.00 ± 9.02 s of SAL treated group) (Fig. 1C). Interestingly, all the pretreatments, including RES, SIR and FLU (114.30 ± 15.40; 97.67 ± 16.90; 122.00 ± 15.11 respectively, F [4, 25] = 9.73, p < 0.05) significantly improved this LPS-induced increase in the immobility time.

Similarly, the immobility time in tail suspension test (TST) was also significantly increased by LPS treatment (185.70 ± 17.68 s) as compared to SAL treated group (106.80 ± 14.94 s) (Fig. 1D). Pretreatment of the animals with RES, SIR and FLU was found to produce significant protection against the impact of LPS on TST immobility time (109.70 ± 14.13 s, 94.83 ± 10.12 s and 117.50 ± 9.49 s respectively, F [4, 25] = 6.99, p < 0.05).

### Effect of RES and SIR on oxidative stress markers

**Fig. 2:**
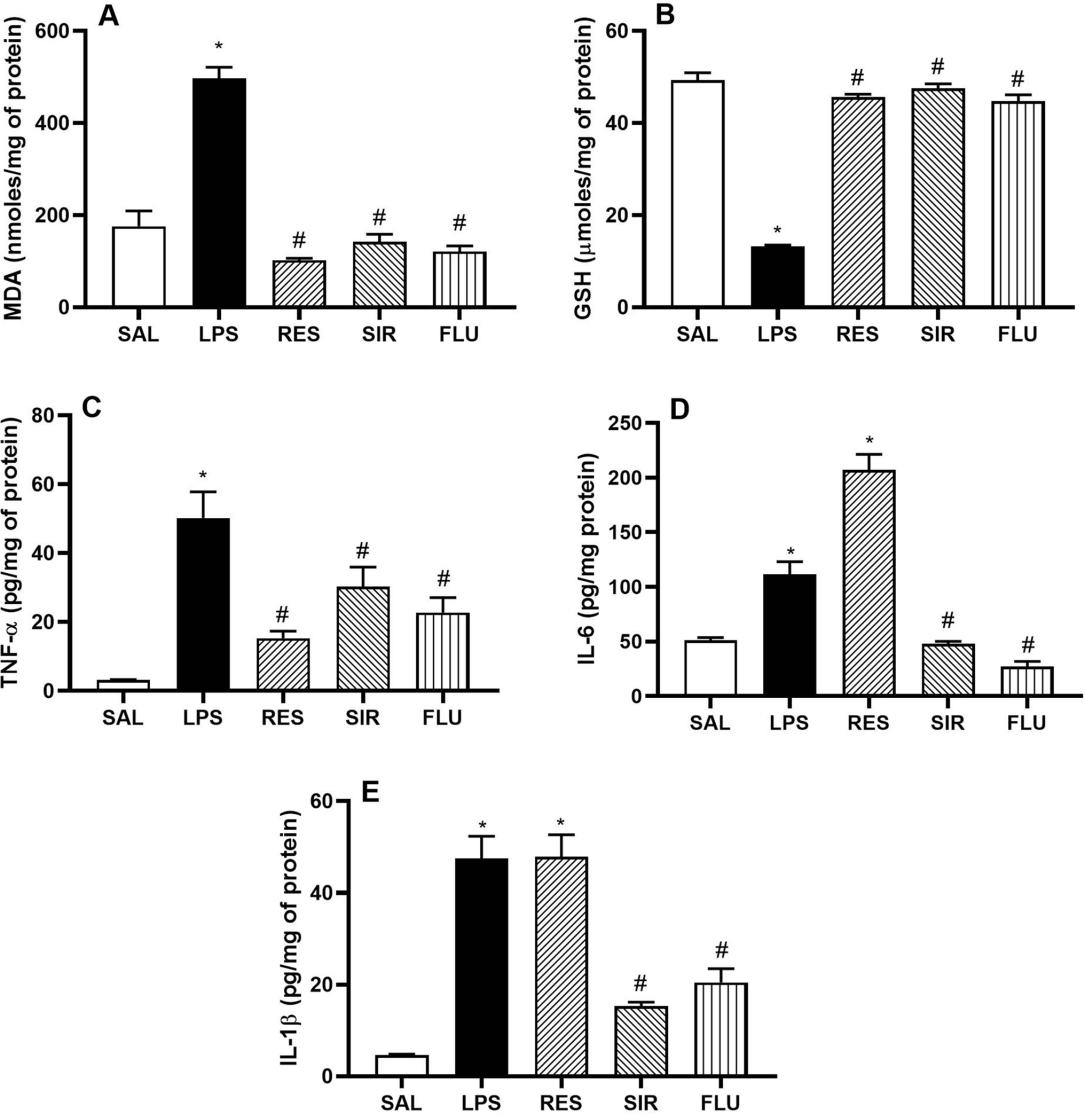
Effect of saline (SAL), resveratrol (RES; 50 mg/kg), sirtinol (SIR; 2 mg/kg) and fluoxetine (FLU; 10 mg/kg) on LPS-induced changes in brain homogenates. MDA levels (nmoles/mg protein) (2A); GSH (µmoles/mg protein) (2B); TNF-α (pg/mg protein) (2C), IL-6 (pg/mg protein) (2D), and IL-1β (pg/mg protein) (2E). *p < 0.05 as compared to SAL group; #p < 0.05 as compared with LPS group

Oxidative stress markers of MDA and GSH were quantified in the brain tissue homogenates of all treatment groups. LPS administration caused a significant increase in lipid peroxidation as quantified by MDA levels (nmol/mg of protein) (496.70 ± 24.38 vs 175.80 ± 33.32 of SAL treated group; Fig. 2A), and it also led to a considerable decrease in total GSH levels (μmol/mg of protein) (13.27 ± 0.26 vs 49.25 ± 1.65 of SAL treated group; Fig. 2B). Pretreatment of the animals with RES (102.20 ± 4.06), SIR (142.20 ± 16.34), and FLU (121.20 ± 11.89, F = [4, 25] = 63.07, p < 0.05, Fig. 2A) offered significant protection against LPS-induced lipid peroxidation. Similarly, the GSH levels were also preserved by pretreatment with RES (45.64 ± 0.62), SIR (47.55 ± 0.99), and FLU (44.80 ± 1.30, F [4, 25] = 194.10, p < 0.05, Fig. 2B).

### Effect of RES and SIR on brain inflammatory markers

Acute administration of LPS significantly increased the levels of proinflammatory cytokines including, TNF-α (50.23 ± 7.60 vs 3.14 ± 0.18 pg/mg protein of SAL), IL-6 (111.50 ± 11.47 vs 51.23 ± 2.49 pg/mg protein of SAL), and IL-1β (47.46 ± 4.93 vs 4.62 ± 0.23 pg/mg protein of SAL, Fig. 2A and 2B). Pretreatment of the animals with RES (15.27 ± 2.08 pg/mg protein) significantly reduced the TNF-α levels, however, both IL-6 (207.20 ± 14.07 pg/mg protein) and IL-1β (47.86 ± 4.81 pg/mg protein) stayed significantly elevated with RES pretreatment. On the other hand, pretreatment with SIR and FLU significantly reduced TNF-α (30.34 ± 5.61 and 22.78 ± 4.35 pg/mg protein respectively, F [4, 20] = 18.60, p < 0.05, Fig. 2C), IL-6 (48.02 ± 2.02 and 27.21 ± 4.51 pg/mg protein respectively, F [4, 21] = 103.8, p < 0.05, Fig. 2D), and IL-1β (15.26 ± 0.94 and 20.48 ± 2.99 pg/mg protein respectively, F [4, 20] = 46.68, p < 0.05, Fig. 2E).

## Discussion

Peripheral administration of bacterial endotoxin (LPS), in animals leads to a biphasic response in both behavioural and biochemical parameters. The acute phase of “sickness behaviour” peaks within 3–4 h of LPS administration and is expressed by a group of symptoms, including anhedonia, slowness in initiation of movement, decreased mobility, exploration and grooming, hunched posture, and hyperalgesia (Painsipp et al. 2011; Berk et al. 2013). In our study, a single administration of LPS in animals led to a significant reduction in both horizontal and vertical activities, as indicated by the significantly reduced number of line crossings and rearing in the open field arena. Furthermore, the immobile state was considerably increased in both FST and TST. Both RES and SIR significantly improved the spontaneous locomotion in LPS treated animals, as the number of line crossings and rearing were significantly increased. Moreover, both pretreatments significantly reduced the immobility time in both FST and TST. These behavioural changes correlated with the neuronal pro-inflammatory cytokines (TNF-α, IL-6 and IL-1β), and oxidative stress markers (MDA and GSH) changes.

LPS acts as a ligand for toll-like receptors (TLR 2 and 4), and it causes the translocation of nuclear factor (NF-κB) by dissociation of inhibitory protein κB (IκB). This initiates a cascade of events leading to activation of immune and inflammatory systems, including expression of cytokines and chemokines, cell proliferation and migration (Yang et al. 1998; Kim et al. 2012; Salt and Palmer 2012; Búfalo et al. 2013). Intraperitoneal injection of LPS compromises the integrity of the blood brain barrier (BBB) by interfering with the physical barriers through damage to endothelial junctions and glycocalyx damage (Wiesinger et al. 2013; Varatharaj and Galea 2017). In our study, LPS produced a significant increase of tested brain cytokines, namely TNF-α, IL-6 and IL-1β. These results correspond with our earlier studies (Mudgal et al. 2019, 2020) where we have shown that the levels of both TNF-α and IL-6 peak rapidly in the plasma and producing a neuroinflammatory state in the brain.

RES has been shown to activate SIRT1 and thereby reducing NF-κB activation by deacetylating p65 subunit (Moon et al. 2013; Jiao and Gong 2020). The most interesting finding of our study was that at the employed doses and schedule, RES reduced the oxidative stress markers and TNF-α in a potent manner, however, there was no noticeable effect of RES on LPS-induced increase in IL-6 and IL-1β. In coherence with these results, it has been shown that in LPS-activated peripheral blood leucocytes, RES produces an overall inhibitory effect on cytokines and chemokines production only during un-stimulated conditions. Where, these pro-inflammatory mediators are secreted in low concentrations as compared to those produced by LPS-stimulated conditions (Richard et al. 2005; Schwager et al. 2017). Further to this, more evidence supports that the production of IL-6 and IL-1β is enhanced by RES in a concentration dependent manner (Schwager et al. 2017). Ex vivo RES treated peripheral blood mononuclear cells from osteoarthritis patients produced higher IL-6 levels in a dose-dependent manner. These results indicate that IL-6 expression might be particularly controlled by SIRT1 (Wendling et al. 2013). Similarly, activation of SIRT1 causes elevation of IL-6 and TNF-α and marginally altering the immune responses in vitro, however, the impact of SIRT1 inhibition or activation on the function of other immune cells remains unclear (Mourits et al. 2021). It is noteworthy that this study involved an acute dosing of RES. Furthermore, IL-1β and IL-6 contribute to Th-lymphocyte differentiation and function, and high levels of these cytokines would prime for adaptive immune response (Mauer et al. 2014). Therefore, chronic treatment with RES is essential before any conclusive statements can be delivered.

Another important finding of this study was that SIR, a nonselective SIRT inhibitor was able to ameliorate LPS-induced upregulation of pro-inflammatory cytokines. Our findings are not exactly in coherence with some of the existing studies where SIRT1 inhibition is involved as a contributing factor for various pathological conditions (Orecchia et al. 2011). We here propose that the effect of SIRT inhibition is dose and regimen dependent, where low and acute dosing of SIR reduces the neuroinflammatory impact of LPS administration. Interestingly, Lugrin et al. (2013) have shown that both SIR and its structural analogue, cambinol impaired the production of IL-6, and TNF-α from macrophages stimulated with LPS. It was suggested that selective SIRT1 and/or SIRT 2 inhibition alone was not effective in reducing the production of these pro-inflammatory cytokines, and SIR could be exhibiting these properties by targeting more than just SIRT1 and SIRT2 (Lugrin et al. 2013). Furthermore, acute pretreatment with SIR at the comparable doses (2.5 and 5 mg/kg, i.p.) has shown to significantly reduce neutrophil elastase induced paw edema and LPS-induced acute lung injury (Tsai et al. 2015). SIR also possess anti-inflammatory properties by affecting the chemokine and adhesion molecule expression (Orecchia et al. 2011) and produces antiproliferative effects in NF-κB p65-independent manner (Fong et al. 2014).

We included FLU as a standard selective serotonin reuptake inhibitor (SSRI) for this study. Apart from its clinically established antidepressant activity FLU has been shown to be effective against LPS-induced inflammation and microglial activation. Moreover, it also reduces inflammatory markers, and oxidative stress along with improvement in the behavioural parameters of FST and TST (Ghosh et al. 2020). Our results are coherent with the existing reports, as FLU significantly reduced the LPS-induced neuroinflammatory and oxidative stress markers, along with normalising the behavioural changes.

This study compared the effects of both SIRT inhibitor (SIR) and activator (RES) is an acute neuroinflammation model of LPS-induced sickness behaviour. We found that the acute effects of SIRT inhibition were more pronounced in reducing the inflammation-induced sickness behaviour in mice. We have substantiated our findings with the existing literature reports. However, it is noteworthy that these SIRT modulators act by various pleiotropic mechanisms, and the effects could further be dependent on the dose, route, schedule of administration and the chemical nature of molecules. Further investigations utilising chronic dosing regimens of these compounds along with their pharmacokinetic profiling would be required to supplement these findings. Moreover, additional studies utilising models of neuroinflammation with multiple injections of endotoxins would be more specific to understand this neuroinflammation-induced neurodegenerative changes.

## Data Availability

The datasets generated during and/or analysed during the current study are available from the corresponding author on reasonable request
